# Novel mitochondrial mutations in the *ATP6* and *ATP8* genes in patients with breast cancer

**DOI:** 10.3892/mmr.2014.2471

**Published:** 2014-08-08

**Authors:** LUDMIŁA GRZYBOWSKA-SZATKOWSKA, BRYGIDA ŚLASKA, JOLANTA RZYMOWSKA, ANNA BRZOZOWSKA, BOLESŁAW FLORIAŃCZYK

**Affiliations:** 1 Department of Oncology, Medical University of Lublin, 20-090 Lublin, Poland; 2 St. John’s Cancer Centre, The Regional Oncology Centre of Lublin, 20-090 Lublin, Poland; 3 Department of Biological Bases of Animal Production, University of Life Sciences in Lublin, 20-950 Lublin, Poland; 4 Department of Biology and Genetics, Medical University of Lublin, 20-093 Lublin, Poland; 5 Department of Clinical Dietetics, Medical University of Lublin, 20-093 Lublin, Poland

**Keywords:** mitochondrial DNA, carcinogenesis, mutations, haplogroups

## Abstract

The role of the mitochondria in the process of carcinogenesis, mainly oxidative phosphorylation, mostly concerns their participation in the production of free radicals and ATP and in the process of apoptosis. The purpose of this study was to detect potential changes in the genes encoding the subunits 6 and 8 of the ATP synthase and their impact on the enzyme’s biochemical properties, structure and function in patients with breast tumors. The tested material was mitochondrial DNA (mtDNA) isolated from specimens of ductal carcinoma (carcinoma ductale) Tp1-2Np0-1Mp0, blood and non-cancerous tissue of mammary gland (control), sampled from 50 patients who had been operated for breast cancer. In the case of missense-type changes in the mtDNA, protein prediction software was used to assess their effect on the biochemical properties of the protein, its structure and function. We identified 8 changes in the *ATP6* gene in 36/50 examined breast cancer cell samples and 5 changes in the *ATP8* gene (10/50). Most of them were homoplasmic changes of missense type. Four of the changes (A8439C, G8858C, C9130G and T9119G) had not been described in the literature before. The identified mutations and polymorphisms, especially those of missense type, can affect mitochondrial functions, especially if the conservative domain of the protein is concerned. Replacement of ‘wild-type’ mtDNA by mutated mtDNA can be an important event in carcinogenesis.

## Introduction

The role of the mitochondria in pathogenesis, especially that related to oxidative phosphorylation (OXPHOS), mainly concerns their role in the process of apoptosis, free radical and ATP production (1). The mitochondrial DNA (mtDNA) contains genes encoding for 2 types of rRNA, 22 types of tRNA and 13 proteins taking part in the process of OXPHOS.

Proteins involved in OXPHOS locate at the inner mitochondrial membrane and constitute the respiratory chain. The mitochondrial chain of electron transport is formed of four large complexes of respiratory enzymes, the complexes I-IV. The proton gradient generated as a result of the transport of reduction equivalents starts the last link of the respiratory chain, which is ATP synthase (F1F0 ATP-asa) also called complex V. The ATP synthase is an enzyme that uses a stream of protons passing through the inner membrane of the mitochondria to synthesize ATP. It consists of a part located on the mitochondrial membrane (F0) and containing a proton channel, and a catalytic component (F1) connected to F0 and locating on the side of the mitochondrial matrix (2). The F0 component contains 3–9 protein subunits (9 in humans) including subunits 6 and 8, encoded by the mtDNA genes *ATP6* and *ATP8*, respectively.

The role of the mitochondria in the process of carcinogenesis is highlighted by the results of recently published studies. These studies revealed a mutation in the nuclear DNA (nDNA) on the fumarate hydratase gene in myoma and kidney cancer, as well as mutations in genes encoding 3/4 subunits of the succinic dehydrogenase belonging to complex II of the respiratory chain, in paraganglioma and pheochromocytoma (3,4).

Zhu *et al* (5) discovered at least one somatic mutation in the mtDNA in 14/15 cases of breast cancer. To date, it is unclear which functions are fulfilled by the mtDNA, and especially the genes encoding proteins involved in OXPHOS, in the context of cancer, although it seems that these genes play an important part in the process. The purpose of this study was to analyze mutations in the sequences encoding subunits 6 and 8 of the ATP synthase and their effect on the biochemical properties, structure and function of the enzyme.

## Materials and methods

### Samples and ethics

The tested material was DNA isolated from specimens of ductal carcinoma (carcinoma ductale) Tp1-2Np0-1Mp0, blood and non-cancerous tissue of mammary gland (control). The specimens were collected from 50 patients who had been operated for breast cancer. The patients had received no chemotherapy or hormone therapy and were all perimenopausal. All participants provided informed consent for the use of their biological material (blood taken for routine laboratory testing and tissues removed during surgery) for research purposes. The Institutional Review Board at the Medical University of Lublin approved this study (approval no., KE-254/141/2009).

### Nucleic acids isolation and polymerase chain reaction (PCR)

DNA was extracted from tumor tissues and the corresponding non-tumor tissues with the DNeasy Blood and Tissue kit (Qiagen, Hilden, Germany). DNA was isolated on the automated nucleic acid extraction system QIACube (Qiagen). DNA samples were qualitatively and quantitatively assessed by electrophoretic separation on an agarose gel and spectrophotometric measurements of sample absorbance in a BioPhotometer spectrophotometer (Eppendorf, Hamburg, Germany), respectively.

The isolated DNA was used to amplify fragments of mitochondrial genes: mitochondrially encoded ATP synthase 6 (*ATP6*) and *ATP8*. The PCR primers for both: ATP8 and ATP6 (868 bp fragment from 8,312 to 9,280 bp position) were: forward (F), 5′-CCACTGTAAAGCTAACTTAGC-3′, position: 8,312–8,332 bp and reverse (R), 5′-GTTAGG GGTCATGGGCTG, position: 9,263–9,280 bp. They were designed based on sequences available from the complete mitochondrial genome of *Homo sapiens* (GenBank accession no., AB055387) using the Primer3 program (http://frodo.wi.mit.edu/). The amplification products were visualized in a 2% agarose gel. The amplicons were then sequenced on both strands using the Applied Biosystems^®^ GeneAmp PCR system 9700 and the BigDye^®^Terminator Cycle Sequencing kit (both from Applied Biosystems, Foster City, CA, USA). The samples were subsequently purified on CentriSep™ columns according to the manufacturer’s protocol or precipitated with ethanol and sodium acetate according to the protocol of the BigDye kit manufacturer (both from Thermo Fisher Scientific). Extension products were separated on the Applied Biosystems^®^ ABI 377 automated sequencer (Applied Biosystems).

### Prediction of changes at the protein level

The impact of the identified mutations on the physical and biochemical properties of the corresponding peptides was assessed as follows: The probability of deleterious mutations, i.e. a functional effect of non-synonymous (amino acid-changing) protein-coding single nucleotide polymorphisms (SNPs), was determined using the PANTHER classification system (6), which estimates the value of substitution position-specific evolutionary conservation (subPSEC) and the probability of a deleterious effect on protein function (P_deleterious_, probability of functional impairment) on the basis of alignment of evolutionarily related proteins. SubPSEC=−3 was used as the cut-off. A SubPSEC=−0.3 corresponds to 50% probability that the SNP will have a negative impact on the function of the protein (P_deleterious_=0.5).

The presence of transmembrane helices in the studied proteins was predicted using the TMHMM 2.0 program (7). The spatial structure of the proteins corresponded, and the location and orientation of the α-helices were predicted using the Pfam database (8). Determination of helicity per residue was performed using the Agadir program (9). The grand average of hydropathy (GRAVY) value (10) and the theoretical isoelectric point (pI) were calculated using the software program tool ProtParam (11). Conservation data were downloaded from the ConSurf-DB (12). The ConSurf-DB is a database that contains precomputed conservation scores for all structures in the Protein Data Bank (PDB). The ConSurf server (13) was used to identify functional regions in the proteins. The probability to observe a given residue in the protein sequence was assessed using a position-specific scoring matrix (PSSM) and the PSSM viewer available from the National Center for Biotechnology Information (http://www.ncbi.nlm.nih.gov/Class/Structure/pssm/pssm_viewer.cgi), using conserved domain (CD) protein alignments. Positive PSSM scores indicate that the given amino acid substitution occurs more frequently in the protein alignment than expected by chance, while negative scores indicate that the substitution occurs less frequently than expected. Polymorphisms were considered to be changes that occured in both blood and tumor cells in the same patient. Mutations were a change characteristic for cancer cells but did not occur in the patients’ blood.

## Results

**Figure 1:**
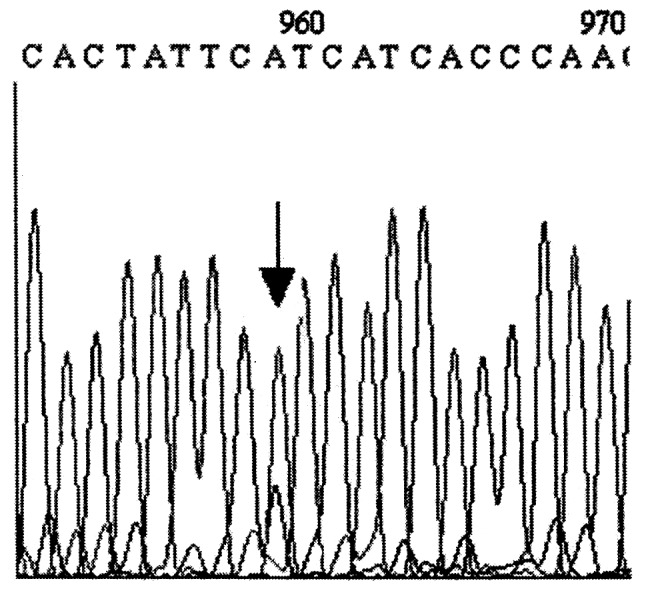
Heteroplasmy in carcinoma cells (transition C8429A).

**Figure 2:**
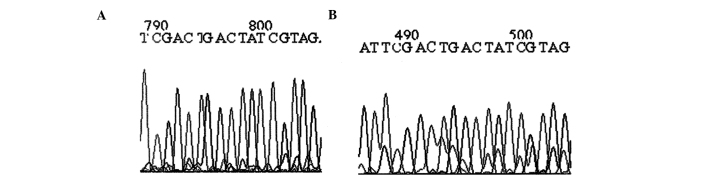
Transitions T9119G and C9130G in the *ATP6* gene in (A) cancer cells and (B) healthy tissue (heteroplasmy in C9130G).

In the gene encoding the subunit 6 of the ATP synthase (*ATP6*), we identified 8 nucleotide changes (Table I) in 72% (36/50) of breast cancer female patients. Five of these (G8557A, G8697A, T8793C, G8854A and A8860G) are defined as polymorphisms in the Human Mitochondrial Genome Database maintained at Uppsala University (http://www.mtdb.igp.uu.se/; Uppsala, Sweden). The remaining 3 have not been previously described in the literature. Heteroplasmy occurred in 2 cases, at positions 8,429 of the *ATP8* gene (Fig. 1) and at position 9,130 of *ATP6* (Fig. 2). At position 9,130, heteroplasmy concerned only the control tissue, and at position 8,429 it concerned only the neoplastic cells.

The polymorphisms G8557A, A8860G and G8854A caused a change in the encoded amino acid (Tables I and II). The polymorphism A8860G is associated with the H2 mitochondrial haplogroup. It was present in 35/50 breast cancer patients. The polymorphism A8860G changing the polar threonine into a non-polar alanine at position 112 concerns α-helix 3 and influences the GRAVY value. According to PANTHER and PSSM analysis, the greatest effect on the function of the protein is exerted by polymorphism A110T, for which subPSEC was estimated at 2.90892 and P_deleterious_ at 0.47725. As shown in Tables III and IV, both amino acid changes L198R and L202V can impact the function of the protein (subPSEC>-3 and P_del_>0.5). The deleterious effect of these mutations confirms their effect on the change of the biochemical properties of the protein, including the percentage of α-helix 6 (Table II). As far as the effect of the L198R mutation on the protein conformation is concerned, TMHMM analysis showed that there is a shift in the amino acids constituting the 4 last intramembrane α-helices. A change in the sections of α-helices occurrence started from α-helice 3.

The L202V mutation does not affect the amino acids that constitute the α-helices. The deleterious effect of this mutation was confirmed by PSSM analysis. A leucine is the preferred amino acid encoded at position 198 (L198R) with PSSM=6, in contrast to arginine (PSSM=−4). At position 202 of the conservative domain of ATP6, the preferred amino acid was leucine (PSSM=6), and it was replaced by valine (PSSM=−1).

In the *ATP8* gene, we identified 5 changes, including the missense mutation A8439C that has not been described before. This mutation concerned a change of the preferred polar amino acid glutamine at position 25 (PSSM=9) into a non-polar one containing a methyl group and an aliphatic side chain, proline (PSSM =−5). The effect of this change on the protein was confirmed by the high P_deleterious_ value (0.83). In addition, the biochemical properties of the protein were affected, as evidenced by the change in the instability index, increasing from 51.40 to 52.51, the grand average of hydropathy, decreasing from −0.336 to −0.332, and the percentage of α-helix, decreasing from 4.8 to 0.51. Overall, these changes indicate that this mutation affects the function of ATP8.

## Discussion

The interest in the role of mitochondria in carcinogenesis was initiated from findings on respiratory deficits in dividing cells, especially in cells with intensive proliferation rates. It is known that respiratory deficits can further impact cell differentiation and can cause neoplastic transformation. Mutations in the mtDNA can be favorable or adaptive, neutral or harmful, i.e. pathogenic. Favorable mutations in the human mtDNA are a result of the adaptation process to the constantly changing external conditions and climate during human evolution. It has been suggested that these adaptive mutations, which have occurred in the mtDNA of ancient human populations during migrations to other continents, are associated with predisposition to certain diseases (4,14,15).

To date, the majority of mutations within the mtDNA have been identified in prostate cancer samples (4,16), and mostly concern the cytochrome *C* oxidase subunit 1 (*COI*). The correlation between mutations in *COI* and prostate cancer showed an increased incidence of prostate cancer among Afro-Americans where mutations occurred frequently compared to Caucasian Americans (16,17). In addition, an increased occurrence of homoplasmic mutations in the *COI* subunit were found in a European population among subjects with prostate cancer in comparison to the controls (11 vs. 7.8%), both in neoplastic cells and lymphocytes (16).

The role of mtDNA somatic mutations in neoplastic progression is still being examined for numerous neoplasms. It is highly probable that mutations in the conservative regions, replication loci, transcription promoters or binding sites for transcription factors may negatively affect the amount of the mitochondrial transcript. On the other hand, mutations in OXPHOS mtDNA genes do not necessarily cause changes in the encoded protein. In pancreatic cancer, among 49 changes in protein-coding regions, 26 did not affect the encoded amino acid. The remaining 23 caused an amino acid replacement in the genes coding for rRNA, NADH dehydrogenase (subunits ND1 to ND5), cytochrome *B*, complex IV of the oxidoreductase cytochrome *C*, and ATP synthase (subunits 6 and 8, and D-loop) (18). In a Chinese population, the *ATP6* gene of osteosarcoma cells harbored mutations in 24/39 patients (19). Furthermore, mtDNA mutations were detected in urinary cancer, and these were more frequent in the respiratory complex-coding regions (20).

In numerous experiments, the identified polymorphisms and mutations within the mtDNA concerned a transition of the types T-to-C and G-to-A, which may imply that they are a result of oxidative stress. The nucleotide guanine, especially in the mtDNA, is preferentially damaged as a result of oxidative stress and the harmful activity of OXPHOS in the nDNA. In our data, 6/13 changes were of type T-to-C and G-to-A, and occurred in 24% of the patients (12/50). In colon cancer, 70% of the identified mutations were a result of reactive oxygen species activity and concerned replacements of type T-to-C and G-to-A (21). The majority of these mutations were somatic and homoplasmic, similarly to another study on ovarian cancer (22). In our study, we found a G-to-A replacement in the *ATP6* gene at positions 8,557, 8,697 and 8,854; these changes are typical changes occurring upon exposure to free radicals.

Somatic cells contain hundreds to several thousand mitochondria, each containing 1–10 gene copies of mtDNA, which, due to its structure (lack of protective activity of histones and the globular, coiled structure of mtDNA), often undergoes spontaneous mutations. It is known that neoplastic cells are of monoclonal origin. It is difficult to explain how, among the many haplotypes of mtDNA, one type is eventually fixed in neoplastic cells, a phenomenon commonly detectable in the form of homoplasmy in the affected tissues (16) and during aging (3). The question is whether the homoplasmic mutations appearing in the mtDNA arise *de novo* or have been inherited. These homoplasmic mutations become apparent with the emergence of clinical symptoms a long period after the mutations’ appearance (when the mutated DNA comes to prevail); these symptoms commonly show slow progression rates. In the examined material in our study, most of the detected changes were of homoplasmic type. In 4 cases (Table I), the transitions were related to both control and tumor tissues and were not detected in blood samples of the patients. It is possible that homoplasmy is associated with mosaicism in these individuals, resulting from paternal mtDNA inheritance, and thus, these mutations may not be associated with the tumor. It can however not be excluded that certain changes occurring in the cells of the tissue from which the tumor originates promote the process of carcinogenesis.

Heteroplasmy occurred in 2 cases in our data: at position 9,130 of the *ATP6* gene, heteroplasmy concerned only the control tissue, while at position 8,429 it concerned only neoplastic cells (Figs. 1 and 2). Heteroplasmy in the control tissue can be a result of changes taking place in the cells of the tissue from which the neoplasm has originated. The mutation at position 9,130 was predicted to affect the function of the protein by both PANTHER and PSSM analysis. It may be that this mutation is beneficial to the function of the mitochondria, which consequently may lead to the elimination of wild-type mtDNA. On the other hand, mutation at position 8,429 has been described in the literature as a polymorphism, and based on PANTHER and PSSM analysis, it is not expected to affect the function of the protein. It is also notable that the replacement L221 was predicted to affect the α-helix (change of helix percentage from 4.80 to 2.28) and the GRAVY value. It was suggested that the mutated mtDNA is replicated at higher rates compared to the wild-type one (1,3). Carcinogenesis involves thousands of successive generations of cells. The process lasts long enough to cause the replacement of ‘wild-type’ mtDNA by mutant mtDNA. It is believed that mitochondrial replication is controlled, and that signaling originating from the functionally changed mitochondria triggers their excessive replication as a result of an improvement in their function (4). This suggests that every mutation in the mtDNA affects their function. However, it is difficult to determine the effects on mitochondrial function exerted by silent mutations, which do not cause a change in the protein. In the examined data herein, two such changes were identified, one in the *ATP8* and one in the *ATP6* gene.

There are two hypothesis concerning the effects of silent mutations: according to the first, silent mutations carry with them ‘unidentified’, difficult to detect mutations that lead to a selective prevalence of the mutated genome and eventually, to the replacement of the wild-type genome with the mutated one. It is also possible that, while the mitochondrial genome does not undergo recombination, mutations are fixed, which results in genetic hitchhiking and causes heteroplasmy and, in the following generations, homoplasmy (4,21,23,24). The second hypothesis suggests that mitochondria control their own replication. In the case of a change in their function, there is increased replication of the ‘mutated’ mitochondrion and eventually, this leads to its prevalence in the cell (4,25).

It appears that besides mutations in the mtDNA, the additional occurrence of polymorphisms is important, which may cause a slight, almost undetectable increase in the production of free radicals. In the examined material, we detected 5 types of polymorphisms. Polymorphism at position 8,860 appeared as many as 33 times in 50 cases. It is connected with the mitochondrial haplogroup H2. In the study of Aikhionbare *et al* (26) concerning ovarian cancer, this polymorphism appeared in 96/102 of the examined tumors. In the remaining patients (17/50) an adenine occurred at this position. Two other polymorphisms seem to be connected with an increased risk of breast and endometrial cancer: the polymorphism at position 10,398 (G-to-A) of the *ND3* gene, associated with the mitochondrial haplogroup N, changes the codon A114T, and is reported to associate with an increased incidence of breast cancer (14,27). Setiawan *et al* (28) did not support this association in their study. On the other hand, the polymorphism at position 16,189 (T-to-C) was associated with endometrial cancer (29). In our previous study (15), we showed the occurrence of polymorphisms in mt tRNA genes in women with breast cancer, including polymorphism 12,308G, which is associated with chronic progressive external ophthalmoplegia (1). It cannot be excluded that polymorphisms may favour the occurrence of selective advantage of the mutated mtDNA.

In the present study, we identified a total of 13 changes in the mtDNA of breast cancer patients, including 4 that have not been described in the literature before. The majority of the changes were homoplasmic, of the missense type. Polymorphisms, especially those of the missense type, can affect the function of the mitochondria, especially if they lie on conservative domains of the mitochondrial proteins. These changes can promote the selective prevalence of mutated mtDNA over the wild-type mtDNA, which may be involved in the process of carcinogenesis. Changes occurring in the mtDNA during carcinogenesis may result from cell adaptation processes to new conditions.

## Figures and Tables

**Table I:** Differences in the *ATP6* and *ATP8* gene sequences between the Cambridge reference (ref.) sequence and the sequences obtained from the mitochondrial DNA of females with breast cancer.

No. of patients (a)	Frequency (mtDB)^b^					Mt. haplogroup	Cambridge ref.	BC cells	BC blood	Healthy breast cells	Amino acid change

	A	G	C	T	del						
*ATP6* polymorphisms											
3 (23,82,84)	21	2,681	2	0	0	H	G8557	G8557A	G8557A	G8557A	A11T
	6	2,698	0	0	0	H2	A8860	A8860G	A8860G	A8860G	T112A
2 (22,24)	0	0	14	2,690	0	M4	T8793	T8793C	T8793C	T8793C	Syn
	6	2,698	0	0	0	H2	A8860	A8860G	A8860G	A8860G	T112A
2 (3,26)	3	2,701	0	0	0	T2	G8854	G8854A	G8854A	G8854A	A110T
	6	2,698	0	0	0	H2	A8860	A8860G	A8860G	A8860G	T112A
22 (2,4–5,10–15, 17,19,21,27–31, 34,36–40,213,81)	6	2,698	0	0	0	H2	A8860	A8860G	A8860G	A8860G	T112A
*ATP6* mutations											
2 (19,27)	128	2,576	0	0	0	JT	G8697	G8697A	G8697	G8697A	Syn
2 (13,15)	0	0	0	0	0	n.a.	G8858	G8858C	G8858	G8858	G111A
1 (28)	0	0	0	0	0	n.a.	T9119	T9119G	T9119	T9119G	L198R
	1	0	2,703	0	0	n.a.	C9130	C9130G	C9130	C9130G	L202V
*ATP8* polymorphisms											
3 (23,82,84)	21	2,681	2	0	0	H	G8557	G8557A	G8557A	G8557A	Syn
*ATP8* mutations											
1 (202)	1	0	2,702	0	0	n.a.	C8429	C8429A	C8429	C8429	L22I
2 (1,30,213)	0	0	0	0	0	n.a.	A8439	A8439C	A8439	A8439	Q25P
2 (4,22)	0	0	5	2,699	0	H11	T8448	T8448C	T8448	T8448C	M28T
1 (81)	1	2,703	0	0	0	n.a.	G8519	G8519A	G8519	G8519	E52K

a Patient’s number;

b data from http://www.mtdb.igp.uu.se.

Mt, mitochondrial; del, deletions; BC, breast cancer; syn, synomymous; n.a., not applicable.

**Table II:** Comparison of protein properties related to the non-synonymous protein-coding SNP in females with breast cancer.

Amino acid change	Theoretical isoelectric point (pI)	Aliphatic index	Instability index	Grand average of hydropathy (GRAVY)	Percentage α helix (start and end of helix)	Region
ATP6						
A11T	10.09	146.37	32.38	0.963	4.67 (5–27)	Transmembrane
T112A					0.57 (98–119)	
A110T	10.09	146.37	31.90	0.963	0.54 (98–119)	Transmembrane
T112A						
G111A	10.09	147.26	33.13	0.984	0.90 (98–119)	Transmembrane
T112A						
T112A	10.09	146.81	32.75	0.984	0.57 (98–119)	Transmembrane
L198R	10.28	144.65	30.67	0.939	14.73 (191–222)	Transmembrane
L202V						
T112A						
L202A	10.09	145.93	31.52	0.965	24.58 (191–222)	Transmembrane
WT	10.09	146.37	32.75	0.963	4.61 (5–27) 0.42 (98–119) 41.36 (191–222)	Transmembrane
ATP8						
Q26P	9.92	78.82	52.51	−0.332	0.51 (1–68)	Transmembrane
M28T	9.92	78.82	46.61	−0.399	3.26 (1–68)	Transmembrane
L22I	9.92	78.82	46.61	−0.388	2.28 (1–68)	Transmembrane
E52K	10.19	78.82	42.66	−0.404	3.24 (1–68)	Mitochondrial matrix
WT	9.92	78.82	51.40	−0.360	4.80	Transmembrane

SNP, single nucleotide polymorphism; WT, wild-type.

**Table III:** Probabilities of functional effects for non-synonymous protein-coding SNPs.

Protein	subPSEC	P_deleterious_	Substitution	MSA position	P_wt_	P_substitution_	NIC
ATP6	−2.18038	0.30584	A11T	61	0.09977	0.28345	3.734
	−2.90892	0.47725	A110T	165	0.18319	0.02946	3.843
	−2.28754	0.32905	G111A	166	0.36732	0.11781	3.843
	−1.43811	0.17338	T112A	167	0.18992	0.15938	3.843
	−3.79435	0.68876	L198R	258	0.24713	0.015	3.931
	−3.91748	0.71453	L202V	262	0.64613	0.03379	3.931
ATP8	−4.58997	0.83061	Q25P	25	0.71968	0.00887	2.049
	−2.74712	0.43711	M28T	28	0.23526	0.02328	2.049
	−0.89758	0.10886	L22I	22	0.22628	0.28675	2.049
	−1.91322	0.25223	E52K	52	0.28393	0.07158	2.049

ATP 6-PTHR11410:SF0-HMM E-value score, 4.9e-92; ATP 8-PTHR13722-HMM E-value score, 6.9e-31. SNP, single nucleotide polymorphism; subPSEC, substitution position-specific evolutionary conservation; MSA, multiple sequence alignment; P_wt_, probability for the wild-type sequence, P_substitution_, probability for the substitution; NIC, number of independent counts.

**Table IV:** Residue frequencies and PSSM scores determined using the PSSM viewer.

Protein (position)	Residue	Raw frequency	Weighted frequency	PSSM score
ATP6^a^				
11	T	0.70	0.62	6
	A	0.11	0.10	1
110	A	0.83	0.73	5
	T	0.03	0.08	1
111	G	0.90	0.83	6
	A	0.09	0.15	2
112	T	0.68	0.76	6
	A	0.32	0.24	2
198	L	0.97	0.95	6
	R	-	-	−4
202	L	1.00	0.99	6
	V	-	-	−1
ATP8^b^				
22	I	0.60	0.52	6
	L	0.13	0.15	2
25	Q	0.98	0.89	9
	P	-	-	−5
28	M	0.06	0.09	4
	T	0.04	0.10	1
52	E	0.78	0.65	7
	K	0.04	0.15	2

a ATP synthase F0 subunit 6;

b ATP synthase F0 subunit 8.

PSSM, position-specific scoring matrix.
